# Supplementary material for: Contribution of peri-urban land use and agriculture to entropy and food of mega-cities: On sustainability, planning by control theory and recycling of organics
Source: PLoS One. 2023 Aug 25;18(8):e0290747. doi: 10.1371/journal.pone.0290747 (PMC10456150; doi:10.1371/journal.pone.0290747)
Supplement: Attachment — Submitted filename: rebuttal-06-04-23.docx [file pone.0290747.s001.docx]

Comments to the remarks of the reviewer:

Reviewer #1: “Do revise and reduce the length of this manuscript for better comprehension“

***Answer: I revised the paper for large (most) parts and think the comprehension is now much better. I reduced the length by 10 p.c. and skipped some explanatory parts in the beginning.***

Reviewer #1: “Include case studies from different region where agriculture is their main economic strength.“

***Answer: I included more references as examples and, in particular referenced to case studies which show the potential but also problems of peri-urban agriculture. As peri-urban agriculture is discussed for many cities in Africa and Asia, yet as option, I only could make reference where authors see scope. Some articles with generalization, which include many case, have been also mentioned.***

Reviewer #1: „Discussion along this line will make this manuscript a good one.“

***Answer: I hope I made clear that there is a need for theoretical foundation of peri-urban and policy tools such as planning.***

Reviewer #2: “The abstract lacks a background theoretical basis on which the whole article is grounded.“

***Answer: I stressed the theoretical background by linking it more to spatial planning as modelling tool. This is now the golden thread of the paper.***

Reviewer #2: “Please revise the abstract to capture some aspects of the conceptual framework of the work and the theory being propounded. As it stands now, it lacks the readings interest that engages readers to want to go further and read the whole article.“

***Answer: I have rewritten the abstract and included a more comprehensive outline what can be expected.***

Reviewer #2: “The introduction to the study is not clear; so many concepts are discussed at the same time: urban-peri-urban-rural land use systems, ecological-economic-social scales entropy, etc making it difficult to grasp the central theme of the background introduction.“

***Answer: The introduction is now more focused and written to what can be expected. I explained terms. However many terms used are subject to special definition and in more detail in the following sections.***

Reviewer #2: “Grammar is very poor and needs to be greatly improved. The whole write-up is saddled with grammatical errors, no coherency in flow of ideas and the ideas are disorganized and disjointed from each other.

***Answer: I revised the paper and checked for Grammar. I hope it is better now. The flow of ideas is clarified by introducing more explanations; though I had to shorten some parts because of recommendations concerning length.***

Reviewer #2: “Because of this, it makes reading of the manuscript difficult and attempt to understand what the write-up is about is fruitless.“

***Answer: I hope there are less gaps in grasping ideas.***

Reviewer #2: “All diagrams are difficult to read and perceive; and to link to the written concepts.

***Answer: For diagrams, I put more explanations in the text. Again it is difficult to write within a limit of pages, even against the request to shorten and then do longer explanations, sorry.***

Reviewer #2: “Authors need to re-organize their thoughts and put in more effort to express them in writing before re-submitting for review consideration.

***Answer: Most thoughts are now expressed along the concept of planning for peri-urban land use. Ideas are several times reiterated. They are explained further in the given contest of each chapter. This is done supplementing previous aspects.***

Reviewer #2: “The topic idea is good, however, the data and writing is poor and do not give enough credit to the topic.

***Answer: Improved.***

________________________________
