# Supplementary material for: Contribution of peri-urban land use and agriculture to entropy and food of mega-cities: On sustainability, planning by control theory and recycling of organics
Source: PLoS One. 2023 Aug 25;18(8):e0290747. doi: 10.1371/journal.pone.0290747 (PMC10456150; doi:10.1371/journal.pone.0290747)
Supplement: Attachment — Submitted filename: rebuttal-29-06-23(4).docx [file pone.0290747.s002.docx]

Comments to the remarks of the reviewer:

**according to the latest call for revision (e-mail: 08-05-23)**

Reviewer #1: Please send for english editing as i found several grammatical errors which reduced the incredibility of this MS.

**which stated that it has been “minor revision” and indicate that the English language and grammar should be checked, I send the article to a professional editor.**

**You will now get the article after revision and the paper has improved also by additional clarifications from my side. This was done along comments you find in the version for correction as well as have gone through the text.**

**Additionally I checked for the references and numbering in the text regarding correct citation.**
